# Behavior Change without Behavior Change Communication: Nudging Handwashing among Primary School Students in Bangladesh

**DOI:** 10.3390/ijerph13010129

**Published:** 2016-01-14

**Authors:** Robert Dreibelbis, Anne Kroeger, Kamal Hossain, Mohini Venkatesh, Pavani K. Ram

**Affiliations:** 1 Center for Applied Social Research, University of Oklahoma, 201 Stephenson Parkway, Suite 4100, Norman, OK 73019, USA; akroeger@ou.edu; 2 Save the Children—Bangladesh, House# CWN (A) 35, Road #43, Gulshan 2, Dhaka 1212, Bangladesh; kamal.hossain@savethechildren.org; 3 Save the Children—USA, 2000 L Street NW, Suite 500, Washington, DC 20036, USA; mvenkatesh@savechildren.org; 4 Department of Epidemiology and Environmental Health, University at Buffalo, The State University of New York, 237 Farber Hall, Buffalo, NY 14214, USA; pkram@buffalo.edu

**Keywords:** handwashing, behavior change, environmental nudges

## Abstract

Behavior change communication for improving handwashing with soap can be labor and resource intensive, yet quality results are difficult to achieve. Nudges are environmental cues engaging unconscious decision-making processes to prompt behavior change. In this proof-of-concept study, we developed an inexpensive set of nudges to encourage handwashing with soap after toilet use in two primary schools in rural Bangladesh. We completed direct observation of behaviors at baseline, after providing traditional handwashing infrastructure, and at multiple time periods following targeted handwashing nudges (1 day, 2 weeks, and 6 weeks). No additional handwashing education or motivational messages were completed. Handwashing with soap among school children was low at baseline (4%), increasing to 68% the day after nudges were completed and 74% at both 2 weeks and 6 weeks post intervention. Results indicate that nudge-based interventions have the potential to improve handwashing with soap among school-aged children in Bangladesh and specific areas of further inquiry are discussed.

## 1. Introduction

Handwashing with soap at key junctures can reduce the risk of enteric and respiratory infections [1,2]; however, improving hand hygiene behaviors, particularly in low-income, resource poor settings, has proven difficult. Traditional approaches to handwashing behavior change are both time and labor intensive and have relied on educational messaging. These messages typically focus on fecal-oral transmission and the recognition of health risks associated with germs [3,4]. Recent interventions have shifted focus to emotional and motivational drivers of handwashing—nurture, disgust, and social affiliation—yet these remain resource intensive [5,6,7]. Regardless of the specific content, handwashing interventions which focus on messaging and/or behavior change communication face noted difficulties in ensuring consistent delivery of high-quality interventions in both domestic and institutional settings [5,7,8,9]. Further, the success of many approaches is markedly similar—handwashing with soap rates among intervention groups of 30%–35% [3,4,6,10]—rates that may be too low to see population-level impacts. Moreover, lasting behavior change from educational and motivational messaging may also be contingent upon repeated reminders or cues to action, relying on conscious decision-making regarding intent and will to change behavior [11].

In high-income countries, “nudging” has gained attention as a means to trigger desired behavioral outcomes. Instead of changing the conscious decision-making process, nudges alter the environmental context in which a decision or behavior is completed [12]. Nudges can take many forms, including environmental cues that engage automatic decision making processes that are quick and unconscious rather than self-aware, goal-oriented, and controlled decision-making. Successful nudges have reduced food waste by 30% to 50% by not offering trays in cafeterias [13], increased positive recycling behaviors by 46% when footprints led individuals to recycling bins [14], and reduced portion size by serving food in smaller bowls (16%) while increasing perceived food intake (7%) [15].

In this pilot study, we tested the potential for nudge-based interventions to improve handwashing behaviors after toilet use among primary school-aged children in rural Bangladesh. We focused our proof-of-concept study on primary schools in Bangladesh. The school environment presents an opportunity for the efficient spread of enteric and respiratory pathogens, and prevention in schools must be prioritized. Behavior change interventions in schools have resulted in handwashing improvements similar to those seen in domestic settings. Two trials in Kenya—one in rural western Kenya [16], one in urban Nairobi [17]—that both included regular soap provision and hygiene education resulted in pupil handwashing rates following toileting events of 32%–38% compared to 2%–3% of students in control schools. Improving hand hygiene in schools—alone or in combination with other water, sanitation, and hygiene interventions—has been shown to reduce pupil absence [9,18] and pupil illness [19,20]. Evidence also suggests that school-based hygiene interventions may also reduce illness among siblings under the age of five [21]. However, ensuring the consistent delivery of interventions in schools, particular in low-resource settings [8,9,22], remains a challenge.

## 2. Experimental Section

**Figure 1:**
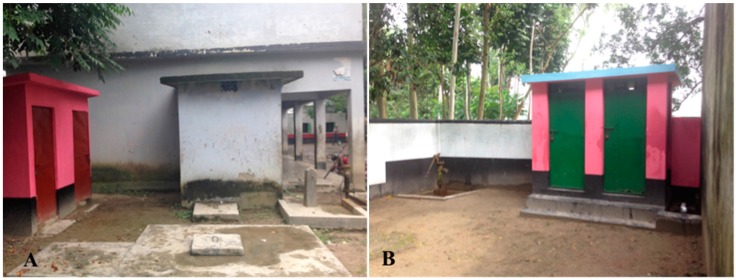
School latrine and water infrastructure prior to nudges in School (**A**) and School (**B**).

**Figure 2:**
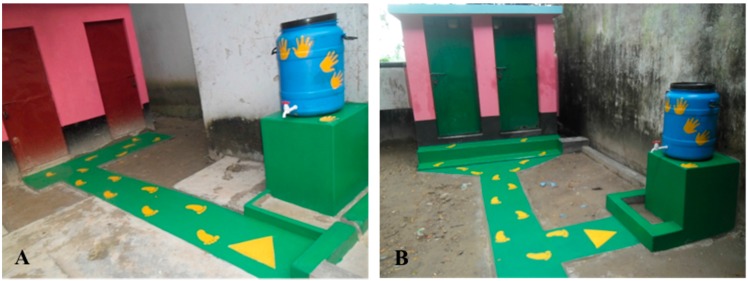
Nudges following final phase of construction, including: dedicated handwashing station, paved paths, and painted foot—and handprints in School (**A**) and School (**B**) (photo taken immediately following construction and prior to soap being made available during the next school day).

In August 2014, we identified two rural primary schools in Bangladesh for our proof-of-concept study. Schools had 220 and 514 students each. Both had on-site water connections (handpumps) and latrines (Figure 1). Prior to the intervention, soap was reported placed at the handpump in a small bowl for students to use during the day, although head teachers reported that this was implemented inconsistently. After assessing site-specific infrastructure, movement patterns, and infrastructure layout, a common set of handwashing (HW) infrastructure improvements and nudges were developed to facilitate handwashing with soap after using the latrine. Infrastructure improvements included the construction of a dedicated location for handwashing (raised cement platform) with a 60 L water container. Two nudges were used at both schools: (1) connecting latrines to the handwashing station via paved pathways that were painted bright colors; and (2) painting footprints on footpaths guiding students to the handwashing stations and handprints on stations (See Figure 2). Each phase of construction—infrastructure, footpaths, and painting—was completed on a single day and observations of pupil handwashing completed the following school day. Construction of each subsequent phase occurred the day after data collection.

During the study, soap was provided to all participating schools and school custodians were instructed to make soap available and refill water storage containers at the start of each school day. No other hygiene promotion activities were included as part of the intervention.

For direct observations, trained staff positioned at discreet locations on school grounds completed structured observations of handwashing practices after leaving the school latrine, specifically if the children washed both hands and whether soap was used. Observations were completed at baseline (prior to construction), 1 day after each infrastructure and nudge installation (HW infrastructure, brick paths, and painting) and 2 and 6 weeks after the intervention was completed. Each observation period consisted of one full school day, typically ranging from 8 am to 3 pm. Observers were allowed to take short breaks during observations during periods when students were in class. Observers were given extra soap to place either at the handpump (at baseline) or at the handwashing station if supplies were not available that day, although this was only necessary during baseline observations.

School principles gave informed consent (*loco parentis*) for participation prior to interventions and data collection. The study was conducted in accordance with standard ethical guidelines for research, and the protocol was approved by the Institutational Review Board at the University of Oklahoma (Protocol 4499).

## 3. Results

Local laborers constructed all infrastructure and nudges using materials sourced on-site in local markets. Hand and footprints were painted using stencils. No special training was required for building or painting. The material and labor costs for the intervention—including water tanks, handwashing platforms, paths, painting, and skilled masons-totaled 161 USD per school.

**Figure 3:**
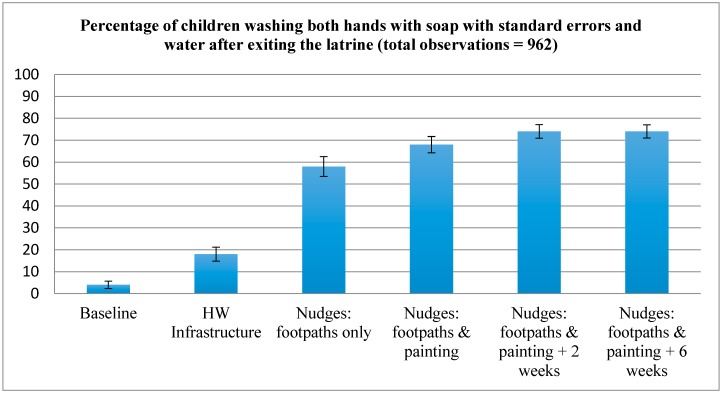
Percentage of children washing both hands with soap following latrine use in two primary schools in rural Bangladesh. HW infrastructure (water tank and raised platform), Nudges: footpath only, and Nudges: footpaths & painting were completed the school day immediately following construction.

Six rounds of data collection yielded 962 observations of children leaving school latrines (Figure 3). At baseline, 4% of children (4 out of 114) were observed to wash both hands with soap after leaving the latrine. After construction of the HW infrastructure, handwashing with soap increased to 18% (26 out of 145). After foot paths connecting latrines to HW infrastructure (the first nudge) were built, 58% of children washed both hands after leaving the latrine (69 out of 119). The day after the final nudge—painting footprints leading from latrines to handwashing stations and adding handprints to the HW infrastructure—68% of children leaving the latrine (108 of 158) washed both hands with soap. Two weeks after the intervention, 74% (151 of 204) washed both hands with soap after leaving the latrine; and after 6 weeks, handwashing with soap remained high (74%; 164 of 222).

## 4. Discussion

This proof-of-concept project provides evidence that simple, low-cost nudges can prompt significant behavior change and result in higher rates of handwashing among school-aged children. Our nudges were simple to install and used local labor and locally available materials without additional training or capacity building. The total intervention costs, 161 USD per school, is significantly less than the cost for educational interventions, estimated at 206 USD for the first month and 53 USD for each subsequent month (Save the Children, internal calculations). Direct comparison of intervention costs should be considered with caution, however. Education-based interventions are typically intended to promote improved handwashing beyond the immediate school environment, thus can have a broader impact on behaviors. If and how nudges inform the creation of new habits that extend beyond the school environment requires further exploration.

There is general recognition that handwashing infrastructure and materials are an important component of the enabling environment for behavior change [23,24]. Infrastructure for handwashing, specifically a dedicated location for handwashing with both soap and water, has been associated with a reduction in the odds of symptoms of respiratory infection [25] and found to complement hygiene education programs [7]. Improved infrastructure alone—the handwashing tank and the raised platform—led to a slight increase in positive handwashing behaviors (14 percentage point increase over baseline); however, this increase is likely not sufficient to create population-level health impacts.

Observed rates of handwashing in our study after nudges were installed are comparable to those seen following intensive educational and/or motivational messaging in both community [3,4,6] and educational settings [16,17]. This was accomplished without any interpersonal communication or educational messaging targeting students or teachers. Nudges may be particularly well suited for educational settings—there are limited external stimuli competing for individual attention, infrastructure serves multiple individuals, and large numbers of people are exposed to the same nudges.

The high rates of observed handwashing at 2-weeks and 6-weeks (74%) after the intervention suggest that environmental nudges can have lasting effects on behaviors. External nudges may require pre-existing knowledge or behavioral intention to successfully trigger desired behavioral outcomes. It is beyond the scope of the study to determine if this latent knowledge of handwashing is due to targeted behavior change interventions or modeling behaviors in the home and other settings. Nudges may present a viable alternative to traditional messaging and behavior change communication programs when handwashing knowledge already exists. The potential synergies of combining the two approaches in school settings (education + nudges) requires further investigation.

Our pilot project was limited to just two schools based on budgetary constraints. Trials with a larger number of participating schools and over prolonged durations are needed in order to fully document the potential and limitations of environmental nudges. Schools with robust water infrastructure were selected in order to concentrate on the immediate environment surrounding handwashing—our intervention did not address underlying issues of water availability and inconsistent provision of materials that limit handwashing in many resource-constrained settings [17]. While our observations indicate that school custodians were active stewards of the intervention, the need to maintain school facilities and provide consistent access to handwashing materials over time must also be considered. Our sustained presence in the schools during construction and during handwashing observations may have altered students’ behaviors through social desirability bias [26] or reactivity [27,28] and teachers may have encouraged students to wash hands with soap. In a study in Kenya, reactivity to the presence of an observer increased handwashing rates by approximately 10% compared to covert video surveillance alone [27]. Future studies will utilize video cameras to monitor handwashing behavior to minimize reactivity. There is limited, mixed evidence that repeated observations may result in lasting changes in handwashing behaviors (a “Hawthorne effect”) [29,30], and our sustained presence in schools during infrastructure and nudge installation could have compounded this effect. However, the behavior change observed in students at 6 weeks following implementation is a compelling indicator that nudges can result in significant and lasting handwashing behavior change, although the long-term behavioral impacts require further investigation. The intervention components were maintained through the duration of our six-weeks of follow-up; however the long-term physical durability of our nudges—particularly the wearing of paint on footpaths and functioning of taps—requires further study.

## 5. Conclusions

Our proof-of-concept study has demonstrated that strategic environmental nudges can have a positive and potentially lasting influence on handwashing behaviors among school-aged children. Future studies in a larger number of schools will compare the behavioral impact of nudges against and in combination with motivational and/or educational messaging, explore the long-term sustainability of behavioral impacts, and investigate the potential for nudges to influence handwashing behaviors in domestic and other institutional settings.
